# Design and evaluation of an automated pediatric acute lymphoblastic leukemia registry from clinical data warehouses

**DOI:** 10.1186/s12911-026-03469-2

**Published:** 2026-04-03

**Authors:** Yoona Choi, Jae Yoon Kim, Jung-Hyun Won, Ki Hoon Kim, Yujin Kim, Wona Choi, Bo Kyung Kim, Jae Won Yoo, Nack-Gyun Chung, Jae Wook Lee, Hyoung Jin Kang, In Young Choi, Howard Lee

**Affiliations:** 1 Department of Applied Bioengineering, Graduate School of Convergence Science and Technology, Seoul National University, Seoul, Republic of Korea; 2 Center for Convergence Approaches in Drug Development, Graduate School of Convergence Science and Technology, Seoul National University, Seoul, Republic of Korea; 3 Child Cancer and Rare Disease Project, Seoul National University Hospital, Seoul, Republic of Korea; 4 Department of Medical Informatics, College of Medicine, The Catholic University of Korea, 222 Banpo-daero, Seocho-gu, Seoul, 06591 Republic of Korea; 5 Department of Biomedicine & Health Sciences, The Catholic University of Korea, Seoul, Republic of Korea; 6 Department of Molecular Medicine and Biopharmaceutical Sciences, Graduate School of Convergence Science and Technology, Seoul National University, Seoul, Republic of Korea; 7 Department of Pediatrics, Seoul National University Hospital, Seoul, Republic of Korea; 8 Seoul National University Cancer Research Institute, Seoul, Republic of Korea; 9 Department of Pediatrics, Catholic Hematology Hospital, Seoul St. Mary’s Hospital, College of Medicine, The Catholic University of Korea, Seoul, Republic of Korea; 10 Department of Pediatrics, Seoul National University College of Medicine, Seoul, Republic of Korea; 11 Department of Clinical Pharmacology and Therapeutics, Seoul National University Hospital, 101 Daehak-ro, Jongno-gu, Seoul, 03080 Republic of Korea; 12 Advanced Institutes of Convergence Technology, Suwon, Republic of Korea

**Keywords:** Electronic medical records, Pediatrics, Acute lymphoblastic leukemia (ALL), Registries, Clinical Data Warehouse (CDW)

## Abstract

**Background:**

Real-world data (RWD)–based pediatric acute lymphoblastic leukemia (ALL) registries often face scalability and reliability limits owing to manual data entry and inter-institutional heterogeneity. We evaluated the feasibility of building an automated, high-quality registry using electronic medical records (EMR) and clinical data warehouses (CDWs) to quantitatively assess the Pediatric ALL Automated Registry (PeARL) for (1) automatic extractability (ADE) (2), data quality, and (3) multicenter applicability.

**Methods:**

In this study, we used CDWs to identify patients aged < 18 years with ALL (ICD-10 C91.0) who visited Seoul National University Hospital (SNUH) and the Catholic Medical Center (CMC). between 1990 and 2023. An automated extraction pipeline applied standard mapping, multivariate transformations, and rule-based natural language processing (NLP). Key variables were selected using the Medical Information Standards for Hematologic Cancer. Data quality was evaluated based on 228 rules across five dimensions (completeness, validity, accuracy, uniqueness, and consistency) based on DQ4HEALTH; error rates, defined as the proportion of data elements violating these rules, were calculated before and after quality management.

**Results:**

Overall, 1,609 patients were included (CMC 946; SNUH 663). ADE for key variables was 89.7% at SNUH and 75.0% at CMC; most automations were single-field transformations (SNUH 61.8%; CMC 84.7%), with multivariate transformations and rule-based NLP addressing complex elements. Initial overall error rates were 1.858% (SNUH) and 0.129% (CMC), decreasing to 0.001% at both institutions after quality processes. Differences in CDW structure required additional preprocessing for laboratory and transplant variables, but harmonization was achieved via standardized table specifications and cross-site review.

**Conclusions:**

PeARL integrated standardized mapping, multivariate transformations, and rule-based NLP to enable large-scale automation of a pediatric ALL registry, achieving a 0.001% overall error rate under DQ4HEALTH-based quality management. This clinically guided, standardized framework enables reproducible implementation and scalable automation of pediatric ALL registry construction, supporting multicenter research and the generation of regulatory-grade real-world evidence.

**Clinical trial number:**

Not applicable.

## Introduction

Registries are systematic and structured approaches that collect standardized clinical data for a defined population with a specific medical condition, disease, or exposure in a real-world setting [1]. Registries play a crucial role in supporting drug development by providing essential information for determining the natural history of a disease, selecting sample sizes and study endpoints for interventional and noninterventional studies, and capturing drug use during routine medical practice [2]. Although other real-world data (RWD) sources also facilitate these functions [3], registries offer certain advantages, such as the collection of structured and predetermined data elements and the provision of curated data about a defined population of patients and their corresponding disease courses [4].

In rare diseases with low prevalence, registries can be particularly valuable in supporting drug development efforts [5]. Providing information on natural disease history when conducting randomized controlled trials is challenging because of the scarcity of patients; however, registries can support regulatory decision-making. For example, blinatumomab was approved by the United States Food and Drug Administration for the treatment of relapsed or refractory B-cell precursor acute lymphoblastic leukemia (ALL) using a registry as an external control arm to support the efficacy of the drug reported in a single-arm phase 2 trial [6].

However, conventional registries rely on medical staff to manually enter data into case report forms, which presents several challenges. The process of identifying eligible patients and collecting the required data elements can be time-consuming and burdensome for medical staff and has the potential to introduce errors during manual data transcription [7–9]. Therefore, the automated transmission of data from the original source, such as electronic medical records (EMR), is increasingly needed for registry development [10]. By automating the process, the costs and related burdens on medical staff can be reduced while improving data accuracy and quality [10, 11].

Therefore, we aimed to develop PeARL (Pediatric ALL Registry using eLectronic medical record-based RWD) —a disease registry for pediatric patients with ALL with high clinical relevance and reliability by incorporating automated data extraction from EMR-based RWD. To achieve this, we used EMR-based RWD to develop a process and algorithm for automatically collecting the data required for the registry and evaluated the feasibility of constructing a high-quality registry using an automated approach. Our study findings provide a strong scientific evidence for the development of a high-quality, scalable registry framework suitable for multicenter research and regulatory-grade real-world evidence generation.

## Materials and methods

### Data source and study population

This multi-center registry utilized the EMR databases from two university-affiliated tertiary-care hospitals in Seoul, South Korea: Seoul National University Hospital (SNUH) and the Catholic Medical Center (CMC). In South Korea, pediatric patients with ALL are diagnosed and treated at large, university-affiliated hospitals with specialized pediatric oncology services, where comprehensive diagnostic workup and cancer therapy are delivered within a single institution. Accordingly, the EMR data from each institution reflect longitudinal treatment records delivered within that hospital. We used data that were previously extracted, transformed, and loaded into clinical data warehouses (CDWs) to develop the registry. The CDW of CMC includes clinical records from 2009 onward, whereas the CDW of SNUH contains records from 2000 onward. The data encompassed the clinical records of pediatric patients aged < 18 with ALL (International Classification of Diseases, Tenth Revision code: C91.0) who visited SNUH or CMC at least once from January 1990 to December 2023. Specifically, the patient-level data included demographic, medical condition, drug prescriptions, measurement, surgery, and hospital visit information.

The study population was further defined based on diagnostic confirmation using clinical and pathological information. Patients were required to have evidence consistent with ALL, including bone marrow examination findings (blast ≥ 20%) and immunophenotype classification as B-cell, T-cell, or ambiguous lineage. Immunophenotype was determined primarily using flow cytometry results; when flow cytometry data were not available as structured CDW data, immunophenotype and ALL diagnosis were confirmed through manual review of pathology reports and clinical documentation. Patients with malignancy-related diagnostic codes preceding the first ALL code were individually reviewed and excluded when an alternative primary malignancy was confirmed.

### Selection and classification of registry data elements

The registry data elements were selected and categorized to align with version 1.0 of the Medical Information Standards for Hematologic Cancer (MISHC) source dataset developed by the National Cancer Center Korea and the National Cancer Data Center. The MISHC was established as part of a project to develop standard data elements for cancer-specific database construction, aiming to select high-research-value items for hematologic cancers and set criteria for prioritizing these data elements. The source dataset was designed to collect various diagnostic and treatment information, ranging from the patient’s diagnosis and registration in the database to death.

We excluded the following elements to maintain the integrity and pertinence of the data from the MISHC source dataset: (1) those with no variability in the context of a singular disease (i.e., ALL) (2), those irrelevant to pediatric populations, and (3) those with minimal relevance to ALL based on input from participating pediatric hematology-oncology specialists. Five pediatric hematologist-oncologists on the research team (BKK, JWY, NGC, JWL, and HJK) with extensive clinical expertise in the field provided input regarding the selection and classification of data elements for the registry.

### Designing the registry table specifications

Each data element was mapped to its respective CDW data field at each hospital. Data elements that did not directly correspond to a single CDW data field underwent phenotyping, which involves defining these elements as measurable and observable concepts within the CDW based on clinical terminology. Based on standardized phenotyping, each research team at their respective hospital independently conducted mapping, which included listing laboratory tests, drugs, surgeries, and radiation treatments as codes utilized in their respective CDW databases. The mapping results were cross-reviewed by a counterpart hospital team to ensure harmonization and validation.

A table specification document was generated based on the phenotyping and mapping outcomes for each data element, providing a comprehensive framework that specified attributes (e.g., data type and length) and the NOT NULL status for each data element. It also identified primary keys (PKs) and foreign keys (FKs). The NOT NULL constraint ensures that each data element contains a value for every patient record, preventing it from being left empty. PKs are unique identifiers within a table, whereas FKs establish connections between tables by referring to the PKs of other tables.

### Extraction, transformation, and loading for registry development

We extracted, transformed, and loaded the source data from the CDWs into a registry table format based on our table specifications. Researchers from two different hospitals performed the data extraction separately because of variations in the CDW structure and data fields between the two institutions. The SNUH and CMC downloaded data from the CDW in Excel format and developed a script to modify the formatting to conform data that conformed to the registry table structure; the CMC team’s data from the CDW was stored in SQL format. Although different researchers independently extracted, transformed, and loaded the source data in both hospitals, the Python and PostgreSQL scripts were developed per the table specifications to ensure consistency. All scripts were thoroughly reviewed to verify compliance with the table specifications and assess their appropriateness for registry construction.

Natural language processing (NLP) was used to process laboratory test results that were recorded as free-text or semi-structured data and therefore directly analyzable as discrete variables. This primarily applied to specialized cytogenetic and molecular assays that include interpretive information within narrative result fields. For example, fluorescence in situ hybridization (FISH) results documented as text strings (e.g., “nuc ish(CDKN2Ax0,D9Z5 × 2)(170/300)”) were parsed to identify clinically meaningful abnormalities such as gene deletions (e.g., CDKN2A deletion), and chromosome analysis reports recorded in narrative formats (e.g., “43(X, Y) …”) were processed to extract chromosome counts for determination of ploidy status. We adopted a rule-based approach rather than complex machine learning–based NLP methods to enhance efficiency, given the semi-structured nature of the text results in both hospitals. We defined a custom Python function for each laboratory test type tailored to parse and extract relevant information from the unique structure and content of free-text reports. Simultaneously, PostgreSQL logic was developed to handle textual and numerical data systematically, converting them into standardized formats suitable for storage, querying, and analysis. Using this approach, text-based laboratory results were processed not only to extract individual discrete laboratory data elements defined in the registry, but also to derive higher-level clinical variables. Specifically, immunophenotype was captured from flow cytometry reports, and cytogenetic information was captured from fluorescence in situ hybridization (FISH) reports.

### Automated data extraction (ADE) assessments

To assess the relevance of our registry data, we evaluated the extent to which key data elements could undergo ADE. Key data elements were defined as those selected for inclusion in the registry based on MISHC’s source dataset, as determined by the researchers. The degree of automation was quantified using the ADE proportion (Eq. 1), calculated as the proportion of key data elements that can be automatically extracted and processed relative to the total number of key data elements in each table.

1 $$\begin{gathered} \:ADE\:Proportion\left( \% \right) = \hfill \\ \frac{{{\mathrm{Number}}\:{\mathrm{of}}\:{\mathrm{automated}}\:{\mathrm{elements}}}}{{{\mathrm{Total}}\:{\mathrm{number}}\:{\mathrm{of}}\:{\mathrm{key}}\:{\mathrm{data}}\:{\mathrm{elements}}\:{\mathrm{per}}\:{\mathrm{table}}}} \times \:100 \hfill \\ \end{gathered} $$

To obtain the ADE proportion, each key data element was classified based on whether it could be extracted using an automated process. Elements eligible for ADE belonged to one of three categories and contributed to the calculation of the ADE proportion: (1) direct transformation: data that can be retrieved directly from the CDW and mapped to registry items through one-to-one direct mapping or basic code–based transformations; (2) multivariate transformation: data that require pre-processing before extraction, such as handling one-to-many or many-to-many relationships. In most cases, this process requires phenotyping to appropriately define and structure the extracted data; (3) NLP extraction: data extracted using NLP techniques.

Elements not eligible for ADE were those requiring manual collection (i.e., automated methods could not be applied) or that were unavailable in the CDW (i.e., not included in the CDW), making automation infeasible. Accordingly, successful ADE was defined as cases in which the required data were available in the CDW and could be automatically extracted and transformed into a standardized, analyzable variable without manual chart review.

### Data quality assessments

To consistently maintain high-quality data, we implemented a quality management process to ensure that the collected registry data were consistently aligned with their intended purpose, thereby enhancing their long-term usefulness and sustainability.

To evaluate the data quality, we developed 228 quality assessment rules based on the five dimensions of completeness, validity, accuracy, uniqueness, and consistency derived from the DQ4HEALTH model developed by Kim et al. [12]. Furthermore, an SQL-based data diagnostic program was developed to systematically apply these quality rules and quantify data quality levels using error rates as quantitative indicators. Overall and indicator-specific error rates (Eqs. 2 and 3, respectively) were calculated to clearly define the data quality levels.

  

2 $$\begin{gathered} \:Overall\:Error\:Rate\:\left( \% \right) = \hfill \\ \frac{{Total\:number\:of\:error\:data}}{{Total\:number\:of\:data\:in\:the\:table}} \times \:100 \hfill \\ \end{gathered} $$

  

3 $$\begin{gathered} \:Indicator - specific\:Error\:{\mathrm{Rate}}\:\left( \% \right) = \hfill \\ \frac{{Total\:number\:of\:error\:data\:per\:indicator}}{{Total\:number\:of\:\:data\:per\:indicator}} \times \:100 \hfill \\ \end{gathered} $$

The evaluation was conducted using Level 5 (Quality value: 85–100) as the minimum standard based on the ISO/IEC 25,012 criteria to ensure data reliability [13]. Four clinicians, two registry experts, and four data quality specialists performed a comparative evaluation before and after quality management to assess the ALL registry.

## Results

### Baseline characteristics of patients

In the PeARL registry, a total of 1,609 pediatric patients with ALL were included comprising 663 from SNUH and 946 from CMC. Using the automated extraction approach, key demographic and clinical variables—including age, sex, phenotype, laboratory findings, cytogenetic results, and transplantation status—were successfully retrieved from the CDWs of both hospitals. The overall distributions of these variables were comparable between the two institutions, suggesting consistent data quality and structure across different CDW environments. Table 1 summarizes the baseline characteristics of the registry population.

**Table 1:** Baseline characteristics of patients in the registry

	SNUH *N* = 663	CMC *N* = 946
Sex, *n* (%)		
Male	392 (59.1)	549 (58.0)
Female	271 (40.9)	397 (42.0)
Age, n (%)		
< 10	456 (68.8)	609 (64.4)
≥ 10	207 (31.2)	337 (35.6)
Phenotype, n (%)		
B cell	492 (74.2)	807 (85.3)
T cell	77 (11.6)	120 (12.7)
Mixed	6 (1.0)	19 (2.0)
Data not available	88 (13.3)	
WBC at diagnosis (10^9^/L), Median(IQR)	6.42(2.88–19.70)	4.63(2.24–10.04)
Cytogenetics, n (%)		
Hyperdiploidy	25 (3.8)	21(2.2)
ETV6::RUNX1	114 (17.2)	202(21.4)
KMT2A-rearrangement	30 (4.5)	30(3.2)
BCR::ABL1	20 (3.0)	91(9.6)
Others	161 (24.3)	286(30.2)
Data not available	313 (47.2)	316(33.6)
Hematopoietic Stem Cell Transplantation status, n (%)		
Yes	141 (21.7)	220 (23.3)
No	522 (78.7)	726 (76.7)
Follow-up (months), Median(IQR)	57.76 (9.07–103.46)	71.69 (28.19–118.11)

### Registry development

The MISHC source dataset comprised 222 data elements categorized into six primary categories and 18 subcategories. From this dataset, 157 data elements were selected for the registry, excluding 70 data elements based on the predefined exclusion criteria. Data elements were then restructured into: (1) Person (2), Diagnosis (3), Laboratory tests (4), Drug (5), Surgery (6), HSCT, and (7) Visit. Each primary category was divided into 18 subcategories that form an individual table within the registry. Table 2 provides the definitions of each subcategory.

**Table 2:** Registry category and subcategory definitions

Category	Sub-category	Definition
Person	Basic and health information	Confirms the basic information and health status of patients with ALL collected by medical institutions.
Diagnosis	Diagnosis information	Confirms the disease diagnosis and history of patients with ALL.
Laboratory	Diagnostic, bone marrow, flow cytometry, chromosomal, molecular genetics, spinal fluid, and imaging lab data	Confirms the tests performed for the diagnosis and treatment of patients with ALL.
Drug	Drug information	Confirms the medications used to treat ALL
HSCT	Hematopoietic stem-cell transplantation Information	Confirms the HSCT and Graft-versus-host disease of patients with ALL
Surgery	Surgery information	Confirms surgeries relevant to the patients with ALL
Visit	Inpatient and Outpatient Information	Confirms patient visits with ALL

### ADE assessment

Among the 184 registry variables, a substantial proportion could be automatically extracted: 165 (89.7%) at SNUH and 138 (75.0%) at CMC. Variables with high ADE proportions were mainly found in categories such as surgery and visit, which primarily contained simple data elements (e.g., event name, event code, or date) that could be retrieved through single-field mapping. In contrast, categories such as Person and HSCT showed lower automation feasibility at both institutions, with ADE proportions below 80% (SNUH: 80% and 75%; CMC: 70% and 12.5%), reflecting the need for manual collection or clinician interpretation.

Among automatable variables, single-field transformations accounted for the majority—102 of 165 (61.8%) at SNUH and 117 of 138 (84.7%) at CMC. Multi-variable transformations were applied to 34 (20.6%) and 18 (13.0%) variables, and NLP-based extraction to 29 (17.6%) and 3 (2.2%) variables at SNUH and CMC, respectively. The Laboratory category contained the largest number of variables requiring multi-variable transformation or NLP processing.

The overall extraction patterns were largely consistent between the two institutions, although notable differences were observed in the Laboratory and HSCT categories. In the Laboratory table, the number of automatable variables was slightly higher at SNUH (70) than at CMC (67). At SNUH, 14 variables required multi-variable transformation and 10 required NLP, accounting for about one-third of all automatable items. In contrast, CMC required these additional processes for only six variables (four multi-variable and two NLP; 9%). In the HSCT category, automation feasibility was markedly lower, particularly at CMC, where only 4 of 32 variables (12.5%) could be automatically extracted (Table 3).

**Table 3:** Distribution of automatable and non-automatable data elements by institution

Table name	No. of Total Data Elements	Automatable				Not automatable			ADE proportion (%)
		Direct Transformation	Multivariate Transformation	NLP	Sub-total	Manual Collection	Data Unavailable	Sub-Total	
**Person**	**20**	**6 / 5**	**10 / 9**	**0 / 0**	**16 / 14**	**4 / 5**	**0 / 1**	**4 / 6**	**80 / 70**
Person	20	6 / 5	10 / 9	0 / 0	16 / 14	4 / 5	0 / 1	4 / 6	80 / 70
**Diagnosis**	**16**	**7 / 7**	**7 / 4**	**0 / 1**	**14 / 12**	**1 / 4**	**1 / 0**	**2 / 4**	**87.5 / 75**
ALL	11	3 / 3	6 / 3	0 / 1	9 / 7	1 / 4	1 / 0	2 / 4	81.8 / 63.6
Other diagnoses (non-ALL)	5	4 / 4	1 / 1	0 / 0	5 / 5	0 / 0	0 / 0	0 / 0	100 / 100
**Lab**	**73**	**46 / 61**	**14 / 4**	**10 / 2**	**70 / 67**	**2 / 3**	**1 / 3**	**3 / 6**	**95.9 / 91.8**
Peripheral Blood	7	7 / 7	0 / 0	0 / 0	7 / 7	0 / 0	0 / 0	0 / 0	100 / 100
Bone Marrow	9	6 / 4	1 / 4	2 / 1	9 / 9	0 / 0	0 / 0	0 / 0	100 / 100
Flow Cytometry	10	5 / 9	1 / 0	3 / 0	9 / 9	0 / 0	1 / 1	1 / 1	90 / 90
Chromosome	9	4 / 5	3 / 0	2 / 0	9 / 5	0 / 3	0 / 1	0 / 4	100 / 55.6
Molgen	8	5 / 8	3 / 0	0 / 0	8 / 8	0 / 0	0 / 0	0 / 0	100 / 100
FISH	8	3 / 7	3 / 0	2 / 0	8 / 7	0 / 0	0 / 1	0 / 1	100 / 87.5
NGS	9	9 / 9	0 / 0	0 / 0	9 / 9	0 / 0	0 / 0	0 / 0	100 / 100
Cerebrospinal Fluid	7	3 / 6	3 / 0	1 / 1	7 / 7	0 / 0	0 / 0	0 / 0	100 / 100
Imaging	6	4 / 6	0 / 0	0 / 0	4 / 6	2 / 0	0 / 0	2 / 0	66.7 / 100
**Drug**	**26**	**21 / 23**	**2 / 1**	**1 / 0**	**24 / 24**	**0 / 0**	**2 / 2**	**2 / 2**	**92.3 / 92.3**
Chemotherapy	13	9 / 10	1 / 1	1 / 0	11 / 11	0 / 0	2 / 2	2 / 2	84.6 / 84.6
HSCT	7	6 / 7	1 / 0	0 / 0	7 / 7	0 / 0	0 / 0	0 / 0	100 / 100
Others Drugs	6	6 / 6	0 / 0	0 / 0	6 / 6	0 / 0	0 / 0	0 / 0	100 / 100
**Surgery**	**12**	**11 / 12**	**1 / 0**	**0 / 0**	**12 / 12**	**0 / 0**	**0 / 0**	**0 / 0**	**100 / 100**
ALL Surgery	6	5 / 6	1 / 0	0 / 0	6 / 6	0 / 0	0 / 0	0 / 0	100 / 100
Others	6	6 / 6	0 / 0	0 / 0	6 / 6	0 / 0	0 / 0	0 / 0	100 / 100
**HSCT**	**32**	**6 / 4**	**0 / 0**	**18 / 0**	**24 / 4**	**7 / 28**	**1 / 0**	**8 / 28**	**75 / 12.5**
HSCT	23	4 / 2	0 / 0	18 / 0	22 / 2	0 / 21	1 / 0	1 / 21	95.7 / 8.7
GVHD	9	2 / 2	0 / 0	0 / 0	2 / 2	7 / 7	0 / 0	7 / 7	22.2 / 22.2
**Visit**	**5**	**5 / 5**	**0 / 0**	**0 / 0**	**5 / 5**	**0 / 0**	**0 / 0**	**0 / 0**	**100 / 100**
Visit	5	5 / 5	0 / 0	0 / 0	5 / 5	0 / 0	0 / 0	0 / 0	100 / 100
**Sum**	**184**	**102 / 117**	**34 / 18**	**29 / 3**	**165 / 138**	**14 / 40**	**5 / 6**	**19 / 18**	**89.7 / 75**

Note: Automated data extraction (ADE) proportion was defined as the percentage of key registry data elements that could be automatically extracted and transformed into analyzable variables without manual chart review. Automatable elements include those obtained through direct transformation, multivariate transformation, or rule-based natural language processing (NLP). Elements requiring manual collection or deemed unavailable in the clinical data warehouse were classified as not automatable. For each category, values are presented separately for the two participating institutions, SNUH and CMC, respectively.Abbreviations: ALL, Acute lymphoblastic leukemia; FISH, Fluorescence in situ hybridization; HSCT, Hematopoietic stem-cell transplantation; NGS, Next generation sequencing; NLP, Natural language processing; ADE, Automated data extraction

Values are presented as SNUH / CMC (two participating institutions)

ALL, Acute lymphoblastic leukemia; FISH, Fluorescence in situ hybridization; HSCT, Hematopoietic stem-cell transplantation; NGS, Next generation sequencing; NLP, Natural language processing; ADE, Automated data extraction

### Data quality assessment

Before data quality processing, the error rates were 0.129% (73,325 records) and 1.858% (409,022 records) for CMC and SNUH, respectively. The errors were detected and rectified based on completeness, validity, accuracy, uniqueness, and consistency rules (Table 4). The identified error types and corresponding remediation criteria are as follows:

### Completeness

These errors involved missing critical clinical information, such as test items, test dates, and surgery dates. These were rectified by explicitly marking missing essential information as “NA” or by removing incomplete records.

### Validity

These errors included values exceeding permissible ranges or undefined items in the diagnostic and test data, such as meaningless values (e.g., “27” or “28” when the defined valid range was 1–26). These errors were addressed by excluding invalid records and incorporating additional validation checks for the diagnostic and test data.

### Timeline accuracy

These errors occurred when the date of death conflicted temporally with other critical events, such as diagnoses, tests, or hospital admissions; for example, having a diagnosis or test date of 2012-12-08 but a recorded death date of 2011-12-08. These were corrected by verifying the death dates against official certificates to ensure accurate alignment with clinical events.

### Business rule accuracy

These errors included missing prescriptions or tests within defined timeframes or extended prescription reference dates. For example, patients prescribed leucovorin should receive methotrexate within seven days; cases lacking this record were identified as containing an error. These errors were intentionally left unresolved because of the necessity for a flexible interpretation based on the clinical context.

### Uniqueness

These errors involved duplicate PK (ID) values were resolved by removing and reloading the entire table.

### Relationship consistency

These errors were due to missing patient numbers from external institutions or absent FK indexes risk inaccuracies in the patient master data and rectified by establishing referential integrity through appropriate FK indexing.

After applying the data quality processes, the error rates decreased significantly, to 0.001% (411 records) at CMC and 0.001% (84 records) at SNUH. All errors except those related to business rule accuracy were successfully rectified. The data quality of the ALL registry achieved a reliability of 99%.

**Table 4:** Data quality before and after quality processing

Institution	Dimension	Subdimension	Before data quality implementation			After data quality implementation		
			Total count	Error count	Error rate (%)	Total count	Error count	Error rate (%)
SNUH	Completeness	-	12,752,909	408,036	3.199	13,236,566	0	0
	Validity	Range	1,087,059	902	0.082	1,311,039	0	0
		Format	2,010,738	0	0	2,065,054	0	0
	Accuracy	Timeline	2,020,726	80	0.003	2,075,042	80	0.003
		Business rule	11,839	4	0.033	11,981	4	0.036
	Uniqueness	-	2,065,265	0	0	2,119,581	0	0
	Consistency	Relationship	2,064,602	0	0	2,118,918	0	0
	**Total**		**22**,**013**,**138**	**409**,**022**	**1.858**	**22**,**938**,**181**	**84**	**0.001**
CMC	Completeness	-	32,044,557	4,346	0.013	30,013,671	0	0
	Validity	Range	4,158,978	17,317	0.416	3,753,807	0	0
		Format	5,267,658	0	0	4,941,958	0	0
	Accuracy	Timeline	5,269,045	939	0.018	4,963,940	0	0
		Business rule	16,567	453	2.734	13,826	395	2.857
	Uniqueness	-	5,151,411	1,526	0.029	4,831,780	0	0
	Consistency	Relationship	5,152,799	49,144	0.954	4,830,834	0	0
	**Total**		**57**,**061**,**015**	**73**,**725**	**0.129**	**53**,**349**,**816**	**395**	**0.001**

SNUH, Seoul National University Hospital; CMC, The Catholic University Medical Center

## Discussion

In this study, we successfully developed a retrospective registry for pediatric ALL that comprehensively captured clinically relevant data through automated extraction of EMR-based RWD from CDWs, achieving both broad scope and high data quality. A total of 184 clinically relevant data elements were identified through expert review by pediatric hemato-oncologists, reflecting key variables essential for characterizing disease presentation, treatment, and outcomes. Of these, the majority were successfully extracted through automated processes (165 (89.7%) at SNUH and 138 (75.0%) at CMC). While a large portion of variables could be directly mapped from structured fields (61.8% at SNUH and 84.8% at CMC), a substantial subset required multivariate transformations or rule-based NLP to resolve complex data relationships and maximize extractability. The registry also exhibited high data quality; after applying rule-based validation procedures, the overall error rate decreased to 0.001% at both institutions, corresponding to an estimated reliability of 99%. Collectively, these findings demonstrate that large-scale, automated, and high-quality registry construction is achievable even in a complex pediatric oncology domain [14–16].

Automated extraction performance showed broadly similar patterns across the two tertiary hospitals, although the overall ADE proportion differed between institutions. These differences mainly reflected how clinical data were organized and transferred into each hospital’s CDW. At SNUH, a larger volume of information had been incorporated into the warehouse but often stored as unstructured text such as narrative lab reports, requiring additional processing. In contrast, at CMC, data covered a slightly narrower range but were mostly tabular, facilitating direct transformations and easier utilization. These observations underscore the need for a thorough understanding of each institution’s CDW structure as well as the development of standardized table specifications and transformation rules for successful multicenter registry automation [10, 17–19].

The design and evaluation of our registry closely align with the framework proposed by the U.S. Food and Drug Administration (FDA) for the use of electronic health records in clinical research, which emphasizes two key dimensions: *relevance* and *reliability* [4, 11]. *Relevance* refers to the availability of critical data such as exposures, outcomes, and covariates, while *reliability* pertains to the accuracy, completeness, and traceability of the data [20–24]. In this study, relevance was addressed by defining clinically essential data elements through close collaboration between pediatric hemato-oncologists and data scientists, guided by national standard data element guidelines. To maximize the representation of these clinically essential elements in the registry, the data were systematically constructed using a combination of automated extraction and manual collection to ensure the availability of key exposures, outcomes, and covariates. Reliability was evaluated using systematic, rule-based data quality assessments applied to the constructed registry, enabling evaluation of data accuracy, completeness, and internal consistency. Although our registry was not explicitly developed for regulatory purposes, these design and evaluation strategies are aligned with the FDA’s relevance–reliability considerations and may facilitate the use of EMR-based real-world data for clinical research [21, 25–28].

While previous real-world data registries for leukemia have been developed in countries such as Sweden [29], Denmark [30], and the US [31], few have reported systematic evaluations of data quality, particularly in pediatric populations. The Danish National Acute Leukemia Registry (DNLR), for instance, assessed 30 selected data elements and reported high accuracy ranging from 89.4% to 100.0% through manual chart review [30]. While this labor-intensive approach demonstrated excellent data quality, its scalability is inherently limited. In contrast, our study employed a rule-based data quality framework grounded in Kim’s DQ4HEALTH model, enabling multidimensional assessment of completeness, validity, accuracy, uniqueness, and consistency. Using this framework, the initial error rate in our registry was already low (0.129% at CMC and 1.858% at SNUH), and subsequent refinement of extraction logic reduced it further to 0.001% at both institutions, corresponding to an estimated reliability of 99%. These findings demonstrate that a clinically informed, automated approach can achieve data quality comparable to traditional manual registries while offering markedly superior scalability and efficiency [30, 32–34].

It is important to note that the extremely low overall error rate reported in this study reflects a data element–level metric calculated across a very large number of registry variables, rather than a patient-level error rate. Accordingly, this value should be interpreted as an indicator of overall data quality at scale, not as evidence that all individual patient records are entirely error-free. Although a small number of residual errors may persist, including the possibility that some errors could occur in clinically important variables, such variables were explicitly prioritized during rule design and data quality assessment. The primary objective of this study was not to eliminate all potential patient-level discrepancies, but to demonstrate that a clinically informed, automated framework can achieve consistently high data quality across large, heterogeneous datasets, supporting reliable multicenter real-world evidence generation.

This study has several key strengths. First, it demonstrates that most essential clinical variables for pediatric ALL can be systematically obtained from EMR-based RWD in CDWs through structured mapping, transformation, and selective rule-based NLP, thereby minimizing reliance on manual chart review [35]. Second, by applying a multidimensional data quality assessment framework covering completeness, validity, accuracy, uniqueness, and consistency, we achieved exceptionally low error rates and established a reproducible process for identifying and addressing extraction issues [36, 37]. Third, unlike conventional registries that depend heavily on manual abstraction, our registry was designed with a structured, automated workflow from the outset. This design not only enables extension of the observation period without additional labor but also facilitates adaptation for multi-institutional use, provided that appropriate data mapping to local CDWs is performed [38–40]. However, successful implementation presupposes a minimum level of data infrastructure, including the availability of structured clinical data, established ETL processes, and institutional governance supporting clinician–data engineer collaboration.

Some study limitations should be considered. First, certain clinically important variables—such as phenotype classification, cytogenetic findings, and radiotherapy details—could not be automated because they require expert interpretation of multiple information sources or were absent in the underlying data. In the future, integrating these variables could be feasible through enhanced NLP techniques and by ensuring systematic recording of such information in structured formats [41]. Second, although the registry was implemented across two tertiary hospitals, differences in CDW structures and coding systems between institutions necessitate additional mapping for replication. Standardization of data dictionaries and adoption of common data models could mitigate this challenge [42]. Third, because the registry was designed as a general-purpose resource to capture a broad set of data elements considered important for pediatric ALL—rather than being tailored to a specific research objective—its data quality has so far been evaluated under general rules. As the registry is applied in actual real-world evidence studies or other investigations, further assessments can determine whether the existing quality levels are maintained under study-specific conditions, thereby confirming its robustness across diverse research contexts [21, 43].

## Conclusions

PeARL registry was developed as a comprehensive automated resource for pediatric ALL, systematically capturing a broad spectrum of clinically relevant data from EMR-based real-world data within clinical data warehouses. Through the integration of structured mapping, multivariate transformation, and rule-based NLP, PeARL achieved a high degree of automation and exceptional data reliability, with an overall error rate of 0.001% [44]. These results demonstrate that automation guided by clinical expertise and standardized validation can deliver a high-quality, scalable registry framework suitable for multicenter research and regulatory-grade real-world evidence generation.

## Data Availability

Deidentified individual participant data that underlie the reported results will be made available to qualified researchers upon reasonable request. Researchers are required to submit a detailed research proposal and sign a data use agreement. Data access will be granted to qualified researchers following review and approval by the PeARL Data Access Committee and the institutional review boards of Seoul St. Mary’s Hospital and Seoul National University Hospital.
